# Newspaper Coverage Effects on the Promotion of a Lifestyle Intervention Program

**DOI:** 10.1155/2013/516767

**Published:** 2013-11-11

**Authors:** Sindre M. Dyrstad, Leif I. Tjelta

**Affiliations:** Department of Education and Sport Science, University of Stavanger, 4036 Stavanger, Norway

## Abstract

The study's purpose was to measure the impact of an individually designed lifestyle intervention program on the readers of a regional newspaper. A newspaper with 180,000 daily readers covered a story about three untrained and overweight adults who participated in an individually designed lifestyle intervention program. Their goals were to become physically fit and run a half marathon (21.1 km) after 14 weeks of training. The newspaper published on average three weekly articles throughout the project period, including the weekly training program and a record of the physical improvements made by the participants. The number of hits on the project's web site was recorded. Spin-off effects on the responses of readers were mapped. The project's web site had 25,000 unique weekly hits. Significant spin-off effects included the establishment of training groups which were still active after two years and the launch of a similar project by another regional newspaper. This individually designed lifestyle intervention program was successfully scaled up and reached a large number of the newspaper's readers. The collaboration between a newspaper and exercise researchers could also be adapted to other press media and represents a novel approach to improve participation in physical activities.

## 1. Introduction

It is well documented that regular physical activity (PA) has many health benefits [1, 2] and that there is a dose-response association between PA and all-cause mortality [3, 4]. However, the PA of the majority of adults does not reach recommended levels, although there seem to be relatively stable or slightly increasing levels of leisure-time PA [5]. Only 20% of Norwegian adults meet the recommended daily requirement for 30 min of moderate-intensity activity [6]. Declining levels of work-related activity, transportation activity, and activity at home have resulted in an overall trend of declining total PA [7]. A further development of strategies to improve population participation in PA is therefore important.

The effectiveness of interventions that promote and increase PA has been studied, and there is strong evidence that public health efforts can successfully increase PA [8, 9]. The intervention methods can range from individually tailored advice to population-wide approaches. Wu et al. [10] studied the cost-effectiveness of interventions used to promote PA. The most cost-effective interventions, such as sign to prompt stair use, reached a high number of people using low-intensity (and low-cost) methods. However, the benefit was limited since these interventions were insufficient on their own to significantly increase the proportion of individuals who satisfied PA recommendations. The least cost-effective interventions were high-intensity “individually adapted behavior change” and “social support” programs. These programs had the largest effect sizes (largest increase in MET hours per person per day) but had a higher cost since they required more face to face counseling or interaction compared to more cost-effective interventions.

The typical intervention recruited people into voluntary groups working towards physical activity goals. Progress was monitored, and continuation of activities was encouraged [8]. Many of these individually adapted behavior change programs have much in common in terms of exercise progression, diet, and motivation. If a program similar to those with a large effect size could be scaled up to reach a large number of people, it would be a cost-effective intervention for increasing PA. An increasing interest in health, fitness, and exercise features in newspapers and magazines has made collaboration with media possible. Health and exercise researchers can design interventions that may help to increase PA and public health behavior, and newspapers can cover the stories of the intervention participants. If the intervention could motivate the newspapers' readers to be more physically active, and these articles attract new readers, this is a win-win situation.

We found no published evaluations of collaborations between newspapers and scientists with the aim of increasing PA among readers. Thus, we started the project “From couch potato to half marathon runner in 14 weeks,” which was a collaboration between a quality Norwegian regional newspaper and the University of Stavanger. The participants were enrolled in an intensive lifestyle intervention program for untrained adults, and their goals were to become physically fit and run a half marathon (21.1 km) after 14 weeks of training. The newspaper aimed to reach a new group of readers by publishing the experiences of the participants in this intervention study and to inspire readers to change their lifestyles and become more physically active. The main purpose of this study was to measure the response from readers regarding reports of untrained adults who participated in an individually designed lifestyle intervention program. We also wanted to gain experience of this intervention method and to explore its possibilities to increase participation in lifestyle intervention programs.

## 2. Methods

### 2.1. Design

This study had an explorative design. The nature of exploratory research is to gain experience that will be helpful in expressing relevant hypothesis for more definite investigation. The main challenge of the study was to intercept readers' response to the newspaper articles during the intervention period, since this was unknown.

The study was initiated by the sports desk of the region's largest newspaper. The newspaper wanted to collaborate with the University of Stavanger to design an exercise program and train three sedentary adults. The newspaper's readers were asked to participate in the 14 weeks long intervention study. The inclusion criteria were that participants had a body mass index between 25 and 35, had to be capable of running a distance of 3,000 m in around 20 min, should not have any kind of knee or hips problems, and could not be engaged in regular endurance training. A total of 169 adults (60% women) aged 17–74 asked to be included in the project. A mix of sexes and ages between 30 and 60 years was desirable. At least one woman should have given birth to one or more children. Two women aged 35 and 40 years and one man aged 58 were selected. The two women had three and two children. These three were selected after having interviewed ten persons who fulfilled the inclusion criteria. All three were interviewed twice and found to be highly motivated. The participants followed a training program, which included four exercise sessions each week. Two of the weekly sessions were supervised by one of two project coaches. In addition, a physiotherapist and a nutritional expert provided advice on alternative training and nutrition. The participants were informed that they would become the focus of public interest because they would receive newspaper coverage every week during the project period. The participants provided written informed consent that all the data related to their training and testing could be published in the newspaper and used in a scientific publication.

### 2.2. Newspaper Coverage of the Project

The daily circulation of the newspaper was 63,000 copies, with an estimated readership of around 180,000 (three readers per copy). Before intervention, the newspaper aimed to publish 2-3 articles per week about the project. Total amount of published articles ended at 48, distributed equally throughout a period of 15 weeks and published both in the newspaper and on the project's website. The printed articles were mostly double sided and contained a picture. Once a week the project was covered on the newspaper's front page. Twenty-two articles focused on the testing, training, and progression of the three participants. Seven articles focused on exercise training principles, three articles on nutrition, and four articles on PA and health. Five of the articles focused on a mix of two or more of the mentioned topics. Every Friday, the training program for the coming week was published in the newspaper and on the newspaper's website. The number of hits on the project's website was registered throughout the project period.

### 2.3. Physical Fitness Tests

The maximal oxygen uptake (${\dot{\text{V}}\text{O}}_{2\max}$) of the participants was measured at the start and after 13 weeks of training using a Vmax 29 oxygen analyzer (SensorMedics, Yorba Linda, CA, USA). A treadmill (ELG 2; Woodway GmbH, Weil am Rhein, Germany) was used as an ergometer, and the inclination was 5.2%. The tests were performed using a standard test procedure [11]. The body weight and height were measured prior to each test. The three participants completed a 3,050 m running test around a lake at baseline and after 13 weeks of training. The participants ran the half marathon race after 14 weeks of training.

### 2.4. Training Program

The 14-week training program was based on elite runners training principles [12], which consisted of a weekly mixture of two intensive interval sessions at 90% of HR_max⁡_ and two continuous running sessions at moderate and low intensity. During the first three training weeks, the effective running time for the interval sessions was 15-16 min, that is, 8 times 2-minute jog at a heart rate of about 90% of HR_max⁡_ with 1 min walk recovery. After five weeks, the effective running period during the interval sessions was increased to 20–24 min. The continuous sessions started with one running session of 20 min and the other session as a combination of 40 min of walking and running. Participants were able to perform this as a complete running session from week 4. After week 5, the longest continuous running sessions were increased by 10 min each week until the participants could complete 100 min of continuous running. The shortest continuous session lasted from 40 to 60 min.

## 3. Results

### 3.1. Public Response from the Intervention Project

All articles were published on the project's website. On average, this website recorded 25,000 unique user hits each week throughout the project period. The lead journalist on the project also received an average of 15 questions or comments each week from readers by e-mail.

The newspaper invited readers to engage in six different internet debates about training, PA, nutrition, and other health issues. Between 20 and 35 questions from the readers were answered during each debate which lasted for two hours. On three occasions, readers were invited to join an open and supervised interval training session with the three project participants. A total of 150 readers joined these three training sessions.

The project had two surprising spin-off effects. First, the participants in the three open interval training sessions established a weekly running group. One year after the end of the project, 30–60 participants aged 15–73 (70% women) still met twice each week for an interval running session. A total of 90 different individuals participated in one or more of these training sessions. Second, a regional newspaper in the south of Norway copied the project. This newspaper followed three untrained adults whose goal was to complete a 41 km cross-country skiing competition, the “Hovden Tour,” after 11 weeks of training. Over 390 readers asked to participate in this project. There were over 100,000 hits on the project's website throughout the project period. Two double-page articles were published in the newspaper's sport pages each week during the project period. On three occasions, the newspaper invited readers to join a supervised interval training session. Seventy individuals participated in the first running session, but the number of participants was limited to 60 in two subsequent technical skiing sessions. The cross-country competition, the “Hovden Tour,” was arranged for the first time in the project year. “Three Lakes Race,” which was the half marathon race the three participants competed in, was organized for the sixth time during the project year (2011). The number of runners who finished the race gradually increased from 150 in the first year (2006) to 460 in 2009. In the year prior to the project year (2010), the number of runners who completed the race was 450. In the project year, the number of competitors who completed the race was 937. In the two previous years, there was also a relay class in the race. Each relay team consisted of five runners; each ran approximately four km. The number of teams increased from 11 in 2010 to 28 in the project year.

### 3.2. Effects of Training on the Participants

Throughout 13 weeks of training, all participants experienced a significant increase in their $\dot{\text{V}}{\text{O}}_{2\max}$, a reduction in their BMI, and an improvement in the running test (Table 1). After seven weeks, they competed in a 10 km road race, and they achieved the following running times: 1:05:14 hour:min:s (female 1 or F1), 1:01:44 (female 2 or F2), and 1:02:07 (male 1 or M1). All participants completed the half marathon race after 14 weeks of training, and they achieved the following times: 2:24:54 (F1), 2:20:18 (F2), and 2:15:30 (M1). In their respective age groups, the participants finished 160 of 172 (F1), 34 of 40 (F2), and 160 of 170 (M1).

## 4. Discussion

The main findings of the study were that the press cover of the three former inactive participants, who achieved their goal of completing the half marathon race based on a 14-week-long lifestyle intervention program, resulted in 25,000 weekly unique hits at the project's website. The readers e-mailed questions, participated at internet debates and open training sessions, and established a weekly training group. The project was also copied by another regional newspaper.

An important criterion for success was that the three participants achieved their goals during the lifestyle intervention period. The participants told their stories through the newspaper, and the impact of the project was dependent on their achievements. Thus, great efforts were made to optimize their physical training programs and to increase the consciousness of participants about healthy lifestyle habits. The male participant stated that this project was one of the best things that could have happened to him. He reduced his body weight by 10 kg and normalized his total cholesterol level and blood pressure. He continued to train four times each week after the project and aimed to participate in future endurance runs. One of the female participants (F1) was one of two coaches who established the running group after the project. She now runs 2-3 weekly running sessions and cycles 20 km to and from her job twice each week. She further stated that “*training has become a part of my life. It has a positive effect on my spirit and my level of energy, and I have enrolled for my next half marathon race*.”

The second female runner (F2) had problems with her hips for several weeks throughout the project period. During this period, she began supervised strength training and replaced two of the running sessions with steep walking sessions on a treadmill (10% gradient). The length and intensity of these sessions were the same as those in the recommended running sessions. After the project, this participant said, “I have reduced my body weight by 10 kilograms as a result of changing my way of living, and my goal is to run another half marathon race in the near future.” The story about her use of alternative training during the injury period was published in the newspaper, and it may have been helpful for others struggling with similar injury problems.

Approximately 60% of the 90 participants in the running group, which was established after the open training sessions, had previously been regularly physically active for more than six months. However, none of them had participated in systematic interval endurance training, and they experienced an improvement in their training quality in the running group. Twelve percent of the participants in the running group had previously not been regularly physically active, and they stated that they saw this open training invitation as a good opportunity to start exercising. To keep the running group motivated and to increase the number of participants, one of the two voluntary coaches sent a weekly e-mail to all 90 participants containing information about the next week's training program and encouraging them to bring a friend. They also started a Facebook group to spread information. A study by Reed [13] showed that e-mail intervention campaigns can be successful in increasing the level of PA among women, so this initiative seemed entirely reasonable. The running group received valuable publicity in newspaper reports, and this publicity was important for recruitment. This training group has recently started a walking group since some of the new participants found running too hard and uncomfortable.

A Norwegian study found that 76% of physically inactive subjects, that is, those who had not engaged in regular physical activity at least once every 14 days, wanted to engage in regular PA. The majority of this group reported that they required more initiatives or greater motivation to change their PA habits. Many were also uncertain whether they would be able to start regular PA [14]. These findings suggest that many people need a push to become physically active. The response to the present project was much greater than expected, and it showed that this type of intervention can provide the necessary motivation to increase PA.

The newspaper gave the project high priority with front-page covers and double-page articles. The project received intensive coverage because of high interest among readers prior to the project. A reader survey of 1,000 newspaper readers was conducted a short time before the project commenced and showed that 60% (around 100,000) of the readers were interested in articles about PA, training, nutrition, and lifestyle. It is reasonable to consider that the newspaper's high level of project coverage increased interest among readers. Another important factor that affected involvement in the project was that the project period was sufficiently short to be attractive to readers but long enough to make the participants fit.

A systematic review by Foster et al. [9] indicated that professional advice and guidance with continued support can encourage people to become more physically active in the short to medium term. Therefore, an improvement of the project would have been to provide more follow-up reports, for example, different exercise programs that focus on the maintenance of physical fitness articles, to maintain the interest and support of readers. The idea of “training people for a better life” and publishing this in the media is not new. This approach has been used in many reality shows and other health programs on television and in health magazines. The publication of personal success stories also occurs on a large scale in commercial advertising. The same principle was applied in the present study, and the large numbers of readers who followed the project showed that the subject was of great interest. However, it is of great importance that readers were inspired to make lifestyle changes themselves and that they actually took steps to begin exercising.

We do not know how many of the new competitors in the local half marathon race (the “Three Lakes Race”) were inspired to copy the three project participants. However, it is not unlikely that some of the increase in race participants could relate to the project “From couch potato to half marathon runner in 14 weeks.”

The transtheoretical model, which is also known as the Stages of Change model [15], could be useful for describing the PA level of readers affected by this project. This model includes five stages of readiness for change. Stage one would include the inactive readers who had not considered becoming more active, whereas inactive readers who wanted to be more active were included at stage two. Stage three included those who wanted to become physically active within one month, and many of these would already be engaged in some form of PA. Stage-four individuals had been regularly physically active for less than six months, whereas those who had been regularly physically active for more than six months were at stage five [16]. We did not determine the characteristics of new participants in the “Three Lakes Race.” However, the three project participants finished near the bottom of the results list. So, despite the doubling of participants from the previous year, it is reasonable to consider that the new race participants were more physically fit than our project participants; that is, they were likely to be defined to stage four or five in the transtheoretical model. Based on the level of participation in the running group, it seems that this group may have been the attraction for people who were occasionally physically active but who wanted more structure in their physical training, which is typical for people at stages three and four. However, the walking group, which was recently established, seems to recruit people from stage two. The newspaper articles could have been important for raising awareness about PA and health, which is essential for people at stages one and two in the transtheoretical model. Thus, it seems reasonable to assume that this type of intervention strategy engaged people at stages two to five, although the main effects were on people at stages three and four. 

The cost of the intervention program for the three participants was around US$6,250, which is high for only three people. However, the project had an estimated 100,000 readers and 25,000 unique weekly hits on the website. We also found that the number of participants in the half marathon race increased by around 500, while 90 people joined the weekly running sessions. Thus, the price per participant was low after taking these figures into consideration, regardless of whether the cost was split among 90, 500, or 25,000 participants. An important factor for this kind of intervention is the collaboration with the press. A public health department would probably have been incapable of conducting such a successful program on its own. First, the promotion budget would have been too high. It would have been very expensive to pay for advertising containing 48 articles, most of which were double-paged and several front-page stories. Second, the level of interest may also have been lower if the project had been published as an advertisement in a newspaper.

Several other regional newspapers also took an interest in the project, and one month after the end of our project the leading regional newspaper in Kristiansand, the fifth-largest city in Norway, started a similar project. Compared with the present study, twice as many readers asked to be included in the project. However, this newspaper only had half the number of printed copies in daily circulation. The lead journalist on the project said that they had reached a new segment of readers, and the large number of hits on the project's web page confirmed that this type of reportage was of great interest. Women comprised over 50% of those who wanted to participate in the skiing project and those who joined the three public training sessions. Traditionally, less than 30% of the competitors in public Norwegian cross-country skiing competitions are women. The overrepresentation of women in the half marathon running project and the cross-country skiing project might suggest that this type of lifestyle intervention program appeals more to women.

According to Hagberg and Lindholm [17], the main criteria for judging the success of a lifestyle intervention program are exercise efficacy, population penetration (capacity to recruit participants), and adherence to PA. We emphasized these criteria during this project. First, we established a well-designed training program with personal trainers, a nutritional expert, and a physiotherapist. We measured the physical fitness of the participants and detected significant improvements after the training period, while the participants reported highly positive experiences with this intensive and individually adapted intervention program. Second, the project received a lot of coverage in the newspaper, which ensured widespread publicity and increased the likelihood of recruiting participants. Third, only three of the 169 highly motivated applicants were included in the study, but the adherence to the program was 100%.

We acknowledge that the study had some limitations. First, there was no control group for measuring the response of the readers to the project. We could have conducted a survey of 1,000 readers and included questions on the possible effects of the study. Second, our estimate of 100,000 readers was based on a reader survey prior to the project and not during the project. However, the survey was carried out only two months before the start of the project. Third, we did not interview the new participants in the half marathon race to determine any possible link with the present project. We therefore do not know how many of the new participants were inspired to start as a result of the newspaper*ʼ*s project coverage. A limitation or challenge with this type of intervention is that the general advice provided in a newspaper is not suitable for all readers. It is not possible to provide individually tailored training programs, so the wrong type of activity and training duration/intensity could result in injuries. Internet meetings and public training are two ways of addressing this challenge.

## 5. Conclusions

There is high media interest in publishing health-related reportage, and this opportunity to motivate healthy lifestyles should be explored more intensively. The study presents a new approach to increase participation in a high-cost lifestyle intervention program. From an international perspective, this project has high potential for promoting public health via increased participation in PA. For example, the newspaper USA Today publishes 1.8 million copies each day, so the paper has around 5.4 million readers if we assume three readers per copy. If this paper launched a similar project and received the same level of interest as the current study, almost three million readers might follow this lifestyle intervention program. Thus, there is great potential for recruiting participants to physical activity interventions by working with a newspaper or other large-scale media organizations. A challenge is to ensure that major editorial desks are made aware of this opportunity to acquire new readers or viewers, and hopefully the present study can facilitate this type of collaboration. 

## Figures and Tables

**Table 1:** Maximum oxygen uptake ($\dot{\text{V}}{\text{O}}_{2\max}$), running time for 3,050 m test, and body mass index (BMI) at baseline and after 13 weeks of training for the three participants.

Participants (age)	$\dot{\text{V}}{\text{O}}_{2\max}$ (mL·kg^−1^·min^−1^)			Running time for 3,050 m (min)			BMI		
	Baseline	After 13 weeks	Difference (%)	Baseline	After 13 weeks	Difference (%)	Baseline	After 13 weeks	Difference (%)
F1 (35)	34.3	37.3	8.7	19:05	17:05	−10.5	32.4	31.6	−2.8
F2 (40)	31.2	39.9	27.9	19:25	17:05	−12.0	24.7	21.8	−11.8
M1 (58)	32.4	43.6	34.6	19:30	16:13	−15.8	28.4	25.7	−9.5

F1: female no. 1, F2: female no. 2, and M1: male no. 1.
